# A novel AI-guided and motorized videolaryngoscope aiming to democratize endotracheal intubation

**DOI:** 10.3389/fmed.2026.1744451

**Published:** 2026-02-12

**Authors:** Robert Harutyunyan, Joshua Morse, David Gauthier, Gilles Bronchti, Thomas Hemmerling, Pascal Laferrière-Langlois

**Affiliations:** 1 Division of Surgical and Interventional Sciences, Department of Surgery, McGill University, Montreal, QC, Canada; 2 Department of Anesthesiology and Pain Medicine, University of Montreal, Montreal, QC, Canada; 3 Department of Anatomy, University of Québec in Trois-Rivières, Trois-Rivières, QC, Canada; 4 Department of Anesthesia, McGill University, Montreal, QC, Canada; 5 Maisonneuve-Rosemont Hospital Research Center, Montreal, QC, Canada

**Keywords:** airway management, artificial intelligence, endotracheal intubation, medical device, motorized intubation, videolaryngoscope

## Abstract

**Background:**

Videolaryngoscopy has improved glottic visualization during intubation, but difficulties in tube advancement and hand-eye coordination continue to limit procedural efficiency. These ergonomic challenges contribute to failed intubations even when visualization is adequate. The present study evaluated a novel artificial intelligence guided and motorized videolaryngoscope (AI-VL) designed to reduce manual dexterity demands and operator workload.

**Methods:**

A preclinical validation was conducted using 5 Thiel-embalmed cadavers. Three operators with varying airway experience performed 30 intubations using the AI-VL. Primary outcomes were first-attempt success rate, intubation time, and visible airway trauma. Secondary outcomes included operator workload using the weighted NASA task load index (NASA-TLX) and post-procedural usability Likert questionnaire.

**Results:**

First-attempt success was 100% across all procedures, with a median intubation time of 14.3 s (IQR 11.0–22.8) and no visible tissue injury. The weighted NASA-TLX global workload was 29.1 ± 6.7 (0–100 scale), below the 25th percentile of published medical task norms, indicating low to moderate perceived demand. Mean usability ratings were 4.3 ± 0.6 on 5, with the highest scores for likelihood of clinical adoption and training suitability, 4.7 ± 0.6 and 5.0 ± 0.0 respectively.

**Conclusion:**

In this cadaveric study, the AI-VL preliminarily demonstrated consistent procedural success, short intubation times, relatively low operator workload, and high usability scores. These findings support its potential as a next generation videolaryngoscope and indicates the need for further clinical trials to explore its effectiveness across diverse clinical settings.

## 1 Introduction

Endotracheal intubation, one of the most frequently performed life-saving procedures, remains inconsistent in non-operating room environments. The democratization of this life-saving technique has been a longstanding goal, as delays or failures in securing the airway can result in irreversible hypoxic injury or death (1). Although videolaryngoscopes (VL) have significantly improved glottic visualization compared to direct laryngoscopy, first-attempt success rate performance in non-operating room settings plateaus at 85% in intensive care units (2). Some publications reported performance as low as 22% in emergency departments when difficult airway criteria are present (3). Moreover, the inability to advance the tube despite clear glottic visualization is a phenomenon explicitly recognized in the 2025 International Standard ISO 7376-2 for VLs (4). This “can see but can't intubate” scenario accounts for 53% of VL failures and highlights the need for innovation beyond improved optics (2).

The technical demand of intubation varies widely depending on operator training and environment. This expertise gap becomes even more pronounced in out-of-hospital settings, where paramedics often resort to supraglottic airway (SGA) devices that provide oxygenation but fail to protect against blood or gastric aspiration, a potentially fatal complication (1). While the use of SGAs would rarely be a first option for in-hospital management of a patient at risk of aspiration and could create medicolegal liability, the routine use, tolerance and omnipresence of this device in emergency medical services reflects the absence of technology democratizing intubation. Therefore, a novel intubation device designed to address these critical shortcomings in VLs could downscale the expertise of intubation, mitigate the pitfalls reported in Supplementary Table 1, minimize training requirements, and remain cost-effective for widespread adoption. To directly address the limitations of existing airway management devices, we developed an artificial intelligence guided and motorized videolaryngoscope (AI-VL), formally known as the Divoscope™ (Divocco AI, Montreal, Canada) integrating two key design features. The objective of this study was to evaluate the performance, perceived workload, and usability profile of this device in a cadaveric setting.

## 2 Materials and methods

### 2.1 Study design and setting

We conducted a pilot study at the anatomy laboratory of University of Quebec in Trois-Rivières to evaluate the preclinical performance of the AI-VL using Thiel-embalmed cadavers, which preserve airway tissue pliability and anatomical fidelity. Three operators with varying levels of experience participated: two anesthesiologists (one experienced with the device, one naive to the technology) and one general medical practitioner experienced with the device but without formal anesthesiology training. The project was approved by the local ethical review board *(Sous-comité d'éthique du laboratoire d'enseignement cadavérique)*.

### 2.2 Device description

The novel AI-VL, introduces a motorized insertion system that advances the endotracheal tube through a protected channel via joystick control, thereby eliminating the bimanual coordination required with conventional techniques. Once the VL is aligned with the glottis, the operator uses a thumb-operated joystick, positioned for the left hand as in conventional laryngoscopy, to control the insertion or retraction of the tube. The AI-VL maintains a form factor comparable to standard VLs and weighs approximately 600 g with the disposable blade attached and an endotracheal tube (ETT) inserted. The motor, as well as other critical components, are integrated within the reusable device which transmits energy to the disposable blade through which the ETT is moved. Mechanical energy is transferred through a passive gear interface at the blade-device connection point, allowing the motor to drive the overmolded wheels within the disposable blade. Notably, the primary cost-driving components are housed within the reusable handle, whereas the disposable blade contains no electronic components and relies solely on its mechanical interface for tube advancement. This mechanism enables the intubation blade to remain at low cost despite fundamentally changing the ergonomics of intubation, freeing the operator's right hand for critical maneuvers such as external laryngeal manipulation, head repositioning, or simultaneous use of adjunct equipment. The integrated channel eliminates the need for a rigid stylet, acting as a guide for the ETT, and protecting it from the teeth during the procedure.

### 2.3 AI description

**Figure 1:**
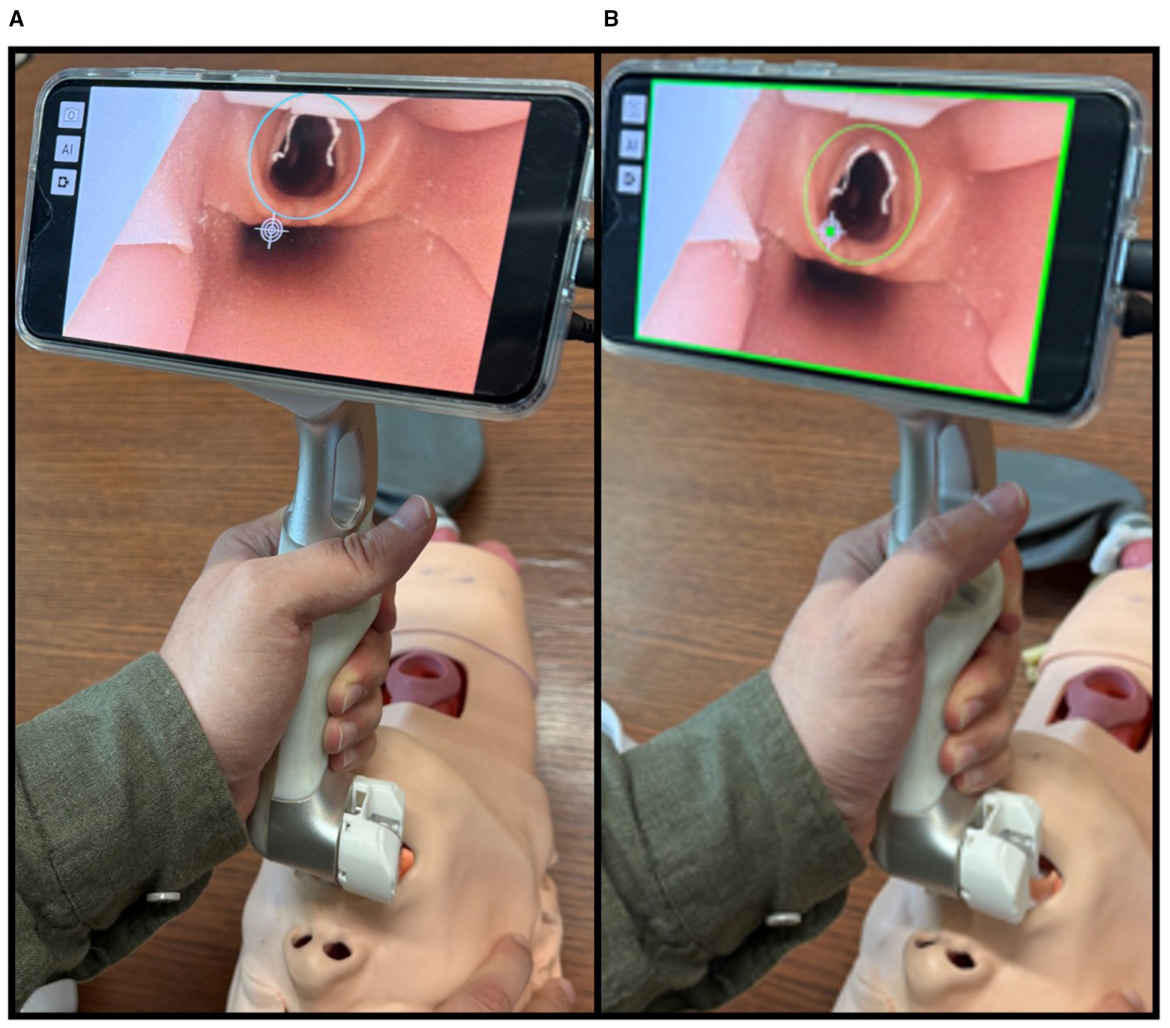
Alignment of the crosshair and glottic opening in a manikin, as identified by the artificial intelligence model on the modular AI-VL. **(A)** Tube alignment not yet achieved. **(B)** Proper alignment, indicating the ETT would enter the trachea if the tube is advanced.

The second innovation integrates computer vision technology that provides real-time decision support. The AI algorithm automatically identifies and highlights the glottic opening while providing visual confirmation when optimal alignment is achieved for tube insertion (Figure 1). The AI module is based on a YOLOv5 convolutional network trained on publicly available VL and intubation video datasets. The operator's focus is to align a fixed crosshair, corresponding to the exit point of the tube, with the AI-derived circle identifying the glottis. Once aligned, the operator activates the joystick to complete the intubation. This AI-based alignment confirmation aims at decreasing the cognitive burden associated with intubation through an intuitive user interface, particularly valuable during high-stress situations where fine motor control and decision-making capabilities are compromised (5). It may also benefit trainees and less experienced operators by simplifying the procedure and accelerating skill acquisition.

### 2.4 Outcomes and data analysis

The primary outcomes were success of intubation on the first attempt, the number of attempts, the overall success, intubation time, and the presence of visible airway trauma. Intubation time is defined as the interval from the moment the operator, positioned at the head of the cadaver with the device in hand, initiated the procedure to visual confirmation of correct endotracheal tube placement. Visible airway trauma was assessed immediately following each intubation attempt by visual inspection of all structures using the videolaryngoscope view and was defined as the presence of macroscopic injury or structural disruption. Following completion of all cadaveric intubations, participants completed two post-procedural assessments to evaluate perceived workload and preliminary device usability. The first consisted of the NASA Task Load Index (NASA-TLX), a validated and widely used multidimensional tool assessing mental, physical and temporal demand, as well as performance, effort and frustration. The second was a 10-item Likert-scale questionnaire comparing the AI-VL with the operator's personal experience with their standard of practice VL across domains of ergonomics, precision, intuitiveness and overall satisfaction. Scores ranged from 1 to 5, with lower scores indicating preference for the conventional videolaryngoscope and higher scores indicating preference for the AI-VL. All results were analyzed descriptively. Continuous variables are reported as means with standard deviations (SD) or as medians with interquartile ranges (IQR), as appropriate. The sample size was estimated assuming a first-attempt success rate of 92%, based on the hypothesis that the reported failure rate of conventional videolaryngoscopy would be reduced by half, requiring 29 intubations to achieve a 10% margin of error with 95% confidence. Given the small sample size and exploratory design, no inferential statistics were performed.

## 3 Results

Each operator performed two intubations per cadaver across five different models, resulting in 30 total intubation attempts. The results demonstrated consistency across all users. First-attempt success rate was 100%, with no failed first-attempt intubations recorded. Median intubation time was 14.3 s (IQR 11.0–22.8 s), which is faster than historical benchmarks for conventional VL typically ranging from 26 to 60 s (2, 6). Performance was consistent among users (Operator 1: median 14.2 s, IQR 11.2–28; Operator 2: 12.0 s, IQR 10.4–13.7; Operator 3: median 20.8 s, IQR 16.2–23.8). While these shortened times can be explained by the reduced number of manipulations, its clinical interpretability must be made in the context of cadaver models, and the absence of situational complexity. Interestingly, some cadavers exhibited difficult airway features. One such example was a restricted mouth opening with inter-incisor gap of 1.5 cm (Supplementary Figure 1). On this specific model, intubation times across the six attempts (median 13.9 s; IQR 11.0–18.1) were close to those for other cadavers. No tissue trauma was observed during any of the procedures, as assessed by the two anesthesiologists (PLL and DG), although this assessment is limited by the cadaveric model.

The weighted global NASA-TLX score for the AI-VL was 29.1 ± 6.7 on the 0–100 scale (Table 1). When interpreted against a meta-analysis of NASA-TLX global workload scores, this score falls below the 25th percentile (~39) across tasks in the medical domain (7). Accordingly, the observed workload is below the reported values suggesting that the AI-VL imposes a low to moderate task load. Amongst the weighted workload dimensions subscales, mental demand (7.3), performance (6.2) and temporal demand (5.3) contributed the most to the overall workload, consistent with the cognitive focus and temporal coordination likely required to visualize and guide the ETT insertion during videolaryngoscopy. Moreover, effort (3.8), frustration (3.3) and physical demand (3.1) remained very low, highlighting the ergonomic comfort and intuitive nature of the joystick-based design.

**Table 1:** Overall weighted NASA-TLX scores.

**Item**	**Original mean (0–10)**	**Weighted mean (0–100)**	SD^*^
Mental	3.0	7.3	5.5
Physical	3.0	3.1	2.8
Temporal	3.0	5.3	4.8
Performance	2.0	6.2	4.0
Effort	2.3	3.8	2.3
Frustration	2.7	3.3	5.8
Overall weighted-TLX (0–100)	–	29.1	6.7

^*^Standard deviation is applicable to the weighted mean.

Usability ratings were favorable with an overall score of 4.3 ± 0.6 on a five-point Likert scale. Operators endorsed clear glottic visualization and intuitive joystick–view coordination and rated the device as highly suitable for training (5.0 ± 0.0), with a high likelihood of future clinical use (4.7 ± 0.6). Likert anchors were defined such that one reflected preference for a traditional videolaryngoscope, three indicated no difference, and five reflected preferences for the AI-VL (Table 2). This level of acceptance, combined with the low workload profile observed on the NASA-TLX, supports that the AI-VL offers a favorable balance between technological complexity and streamlined usability.

**Table 2:** Post-study survey questionnaire results.

**Item**	**Shortened survey question**	**Result (SD)**
1	Clarity and stability of glottic view	4.67 (0.58)
2	Ease of tube advancement	4.00 (1.00)
3	Precision of tube trajectory control	3.67 (1.53)
4	Intuitiveness of coordination between viewing and tube movement	4.33 (1.15)
5	Ergonomic comfort (handle, screen, joystick)	3.33 (1.53)
6	Perceived airway safety/minimization of trauma	3.33 (1.53)
7	Learning curve compared with current method	4.67 (0.58)
8	Usefulness as a teaching/training tool	5.00 (0.00)
9	Overall satisfaction	4.33 (1.15)
10	Likelihood to use or recommend clinically	4.67 (0.58)

All Likert-scale responses ranged from 1 to 5, where a score of 1 reflected a preference for the traditional videolaryngoscope, three indicated no difference between the two devices, and five reflected a preference for the AI-VL.

## 4 Discussion

The preliminary findings demonstrate that AI-guided, motorized videolaryngoscopy can achieve consistent procedural success with minimal perceived workload. The combination of controlled tube advancement and real-time visual alignment support addresses several persistent ergonomic challenges in airway management (Supplementary Table 1). Although the 100% first-attempt success rate was achieved under controlled cadaveric conditions, the uniform performance across operators with differing medical experience levels and different prior exposition to the AI-VL suggests that this technology may reduce the technical threshold required for successful tracheal intubation. The overall NASA-TLX score further supports the device's intuitive usability and efficacious workload profile.

**Figure 2:**
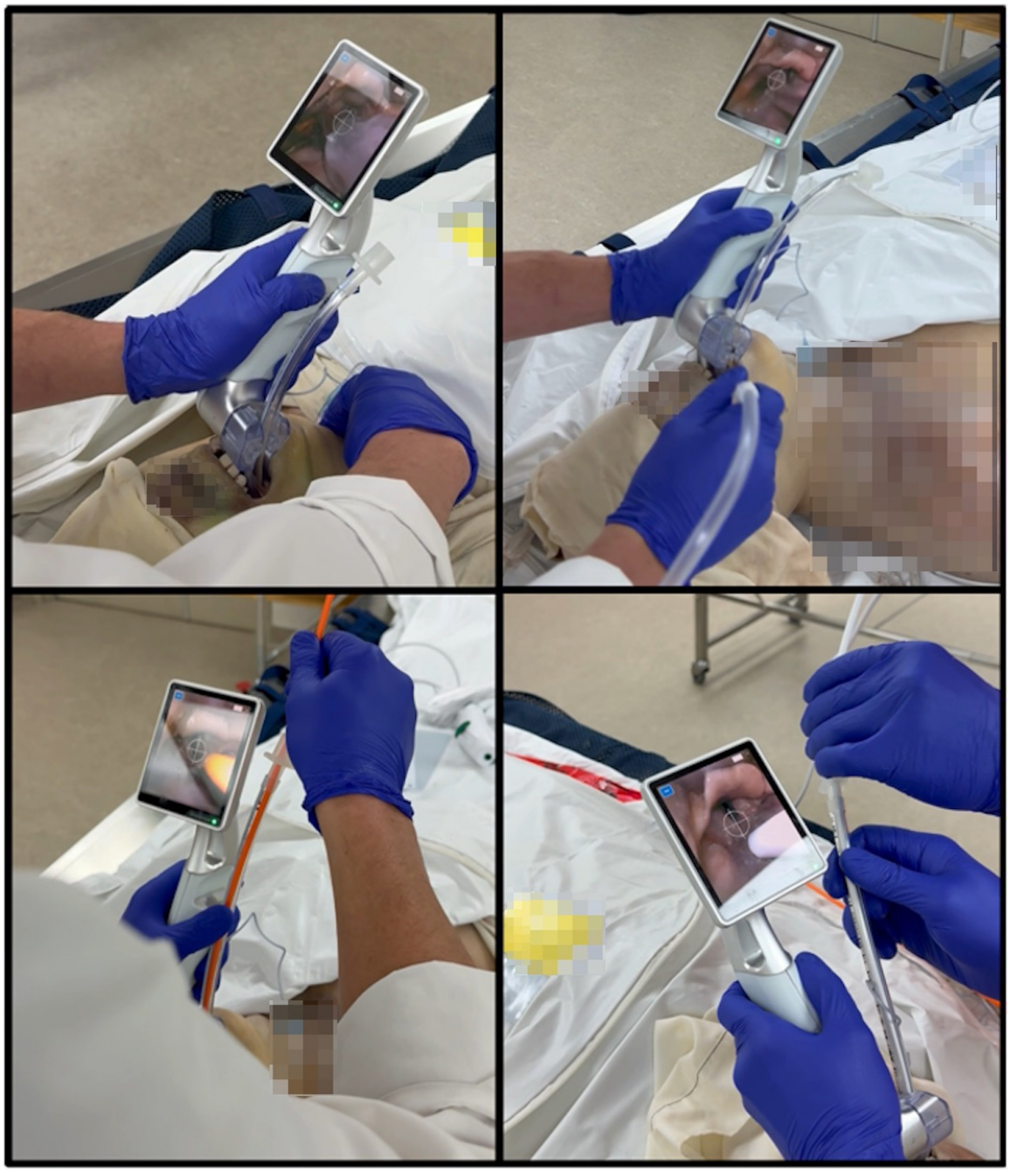
Integrated AI-VL model in use during cadaveric intubation. **(A)** Laryngeal pressure exerted by the right hand of the operator to facilitate intubation. **(B)** Use of suction while simultaneously performing intubation with the joystick. **(C)** Use of a bougie combined with the joystick-based motion of the endotracheal tube. **(D)** Insertion of a flexible fiberscope in the mounted endotracheal tube.

The observed performance and advantages likely result from the integration of both mechanical and cognitive design elements. Traditional VLs require the left hand to maintain laryngoscope position while the right hand simultaneously advances the endotracheal tube, precluding concurrent performance of adjunctive maneuvers by the main operator. These maneuvers are thus performed by a second operator, when available. The motorized tube advancement system fundamentally alters the biomechanics of VL by eliminating the requirement for bimanual coordination during tube passage, without sufficiently impacting the maneuvers to require extensive or supplemental training (Figure 2). With single-handed tube insertion, the operator's right hand is freed allowing external laryngeal manipulation, such as the BURP maneuver (Backward, Upward, Rightward Pressure), to be self-administered, thus optimizing glottic visualization (8). Dynamic patient repositioning, including head extension or lateral rotation, can be adjusted during the procedure based on evolving anatomical visualization. Simultaneous suctioning of blood, secretions, or gastric contents also becomes feasible without interrupting the intubation attempt or releasing the tube. If the operator deemed relevant to use adjunct material, the lumen of the tube remains open throughout the maneuver. Without halting an intubation attempt, the operator can insert and manipulate a bougie with the free hand and still use the motorized system to advance the tube over the guide. Although facilitated by a second operator, hybrid techniques incorporating fiberoptic guidance through or alongside the endotracheal tube can still be performed with simultaneous bronchoscope manipulation and motorized tube advancement. The tactical flexibility to perform complex airway maneuvers independently is operationally relevant, notably in emergency settings where a second provider may be unavailable (9). The reduced need for repositioning and the elimination of a rigid stylet may decrease tissue trauma, pharyngeal laceration, and vocal cord damage but clinical trials will be required to support this further (10).

Beyond these mechanical advantages, the motorized insertion system addresses a critical ergonomic challenge in VLs. Conventional intubation requires simultaneous blade stabilization and tube insertion tasks that depend on fine motor coordination which can often deteriorate in stressful clinical situations (11). By introducing joystick-driven intubation following proper positioning, the AI-VL minimizes the manual dexterity requirements. Supporting this further, the AI decision support component provides real-time cognitive assistance through the automated glottic detection and alignment confirmation that ease mental demands on the operator during the procedure (12, 13). Such cognitive offloading can be especially beneficial for operators who intubate infrequently in addition to emergency situations where stress may impair performance. Early user feedback reported that the static crosshair alignment system with AI-detected glottic highlighting creates a “gamified” interface that provides immediate feedback on positioning accuracy, potentially accelerating the learning curve for novice operators while maintaining reliability for experts (14). Together, the motorized tube advancement paired with AI-driven guidance transforms intubation from a procedure heavily dependent on fine manual dexterity and spatial reasoning into a more intuitive, standardized, technologically assisted process.

Recent airway-management innovation has increasingly focused on smart devices that augment videolaryngoscopy with automation, robotics, and computer vision. Narrative and clinical literature describe rapid growth in AI-assisted interpretation of laryngoscopy images and real-time guidance concepts (15). Notably, a soft-robotic intubation device capable of rapid tube placement in early mannequin and cadaver testing illustrates a parallel pathway toward reducing operator skill demands through automation (16). In contrast, AI-guided videolaryngoscopy systems have largely focused on visual decision support which presents both promise and limitations of purely vision-based assistance (17). Within this landscape, the Divoscope, explored in this trial, aims to address a distinct gap: a step forward from the traditional visualization tool into an integrated guidance-and-delivery aid with meaningful potential in improving first-pass success and reduce operator workload in high-acuity settings.

Several important limitations must be acknowledged in this preliminary evaluation. The cadaveric model, while preserving realistic tissue characteristics, cannot replicate the dynamic challenges of live patients, including active bleeding, secretions, airway reflexes, or patient movement, which may underestimate procedural complexity and intubation difficulty in clinical practice. The study did not test performance in adverse conditions commonly encountered in emergency settings, such as blood or gastric contents obscuring visualization. The workload assessment was conducted on the AI-VL and did not include a comparator device, such as a control VL, limiting the ability to directly contextualize perceived workload relative to a standard VL. Finally, the small sample size and controlled environment limit generalizability to real-world clinical scenarios and may bias results toward favorable performance metrics. Future studies should evaluate learning curves and device performance in live patients across various clinical settings, including emergency departments, intensive care units, and out-of-hospital environments.

In conclusion, the Divoscope represents a prospective advancement in airway management technology, pending clinical validation. By addressing the primary cause of VL failure (i.e., difficulty advancing the tube despite adequate visualization), this innovation may improve intubation success rates across diverse clinical environments and operator skill levels. The preliminary cadaveric data, while promising, highlights the need for comprehensive clinical trials to validate these findings in real-world scenarios. As we move toward an era of increasingly sophisticated medical devices, the integration of AI with mechanical innovation offers confidence for democratizing complex medical procedures. The Divoscope exemplifies this potential by transforming intubation from a skill-dependent procedure into a more standardized, technology-assisted intervention that could ultimately save lives across the continuum of care from operating rooms to battlefield medicine.

## Data Availability

The original contributions presented in the study are included in the article/Supplementary material, further inquiries can be directed to the corresponding author.

## References

[1] Lockey D Coats T Parr M. Aspiration in severe trauma: a prospective study. Anaesthesia. (1999) 54:1097–8. doi: 10.1046/j.1365-2044.1999.00754.x 10540100

[2] Prekker ME Driver BE Trent SA Resnick-Ault D Seitz KP Russell DW . Video versus direct laryngoscopy for tracheal intubation of critically ill adults. N Engl J Med. (2023) 389:418–29. doi: 10.1056/NEJMoa2301601 37326325 PMC11075576

[3] Mosier JM Stolz U Chiu S Sakles JC. Difficult airway management in the emergency department: GlideScope videolaryngoscopy compared to direct laryngoscopy. J Emerg Med. (2012) 42:629–34. doi: 10.1016/j.jemermed.2011.06.007 21911279

[4] Standardization IOf. Anaesthetic and Respiratory Equipment—Laryngoscopes for Tracheal Intubation—Part 2: Video Laryngoscopes. Geneva: International Organization for Standardization (2025).

[5] Lauria MJ Gallo IA Rush S Brooks J Spiegel R Weingart SD. Psychological skills to improve emergency care providers' performance under stress. Ann Emerg Med. (2017) 70:884–90. doi: 10.1016/j.annemergmed.2017.03.018 28460863

[6] Sun DA Warriner CB Parsons DG Klein R Umedaly HS Moult M. The GlideScope^®^ video laryngoscope: randomized clinical trial in 200 patients. Br J Anaesth. (2005) 94:381–4. doi: 10.1093/bja/aei041 15567809

[7] Grier RA editor. How high is high? A meta-analysis of NASA-TLX global workload scores. In: *Proceedings of the Human Factors and Ergonomics Society Annual Meeting*. Los Angeles, CA: Sage Publications Sage CA (2015). doi: 10.1177/1541931215591373

[8] Zeidan A Quintard H Myarta S El-Tahan M. Direct versus video-laryngoscopy: a game-changer for tracheal intubation in critically ill adult patients. Anaesth Crit Care Pain Med. (2023) 43:101316. doi: 10.1016/j.accpm.2023.101316 37865218

[9] Crewdson K Lockey D Voelckel W Temesvari P Lossius HM Group EMW. Best practice advice on pre-hospital emergency anaesthesia & advanced airway management. Scand J Trauma Resusc Emerg Med. (2019) 27:6. doi: 10.1186/s13049-018-0554-6 30665441 PMC6341545

[10] Cook T MacDougall-Davis S. Complications and failure of airway management. Br J Anaesth. (2012) 109(suppl_1):i68–85. doi: 10.1093/bja/aes393 23242753

[11] Nakanishi T Sakamoto S Yoshimura M Fujiwara K Toriumi T. Learning curve of i-gel insertion in novices using a cumulative sum analysis. Sci Rep. (2023) 13:7121. doi: 10.1038/s41598-023-34152-5 37130901 PMC10154413

[12] Topol EJ. High-performance medicine: the convergence of human and artificial intelligence. Nat Med. (2019) 25:44–56. doi: 10.1038/s41591-018-0300-7 30617339

[13] Esteva A Kuprel B Novoa RA Ko J Swetter SM Blau HM . Dermatologist-level classification of skin cancer with deep neural networks. Nature. (2017) 542:115–8. doi: 10.1038/nature21056 28117445 PMC8382232

[14] Simon G DiNardo CD Takahashi K Cascone T Powers C Stevens R . Applying artificial intelligence to address the knowledge gaps in cancer care. Oncologist. (2019) 24:772–82. doi: 10.1634/theoncologist.2018-0257 30446581 PMC6656515

[15] De Rosa S Bignami E Bellini V Battaglini D. The future of artificial intelligence using images and clinical assessment for difficult airway management. Anesth Analg. (2025) 140:317–25. doi: 10.1213/ANE.0000000000006969 38557728 PMC11687942

[16] Haggerty DA Cazzoli JR Wayne MA Winckler CJ Wampler DA Jarvis JL . A soft robotic device for rapid and self-guided intubation. Sci Transl Med. (2025) 17:eads7681. doi: 10.1126/scitranslmed.ads7681 40929248 PMC12812450

[17] Choi J Lee Y Kang GH Jang YS Kim W Choi HY . Educational suitability of new channel-type video-laryngoscope with AI-based glottis guidance system for novices wearing personal-protective-equipment. Medicine. (2022) 101:e28890. doi: 10.1097/MD.0000000000028890 35244042 PMC8896493

