# Supplementary material for: A novel AI-guided and motorized videolaryngoscope aiming to democratize endotracheal intubation
Source: Front Med (Lausanne). 2026 Feb 12;13:1744451. doi: 10.3389/fmed.2026.1744451 (PMC12937148; doi:10.3389/fmed.2026.1744451)
Supplement: Supplementary file 1 [file Table_1.docx]

**Supplementary Table 1.** Major pitfalls associated with endotracheal intubation

| **Pitfalls** | **Comments** |
| --- | --- |
| Can’t position the tube despite good glottic visualization | Most frequent context of failure during videolaryngoscopy.(18) Despite appropriate manipulation, the tube tip remains misplaced. May be attributed to edema or gross deformation. |
| Poor glottic visualization on the screen | A common issue mitigated with the availability of hyperangulated blades.(19) Visualization is typically improved with the BURP maneuver or blade and head repositioning. Can also result from gross deformation of the airway (e.g., trauma). |
| Cuff damage during tube insertion | Contact with teeth may damage the cuff, preventing inflation below the vocal cords and impairing the ability to secure the airway. Tube must be replaced. |
| Secretion or blood in the patient’s airway, impairing view | Visualization of the glottis may be obscured by fluid in the airway. Suctioning is required. |
| Tissue damage during manipulation of the glottis | Prolonged or forceful manipulation of the blade, tube or stylet may result in airway injury (e.g., laceration, bleeding, vocal cord damage). |
