# Supplementary figures and images for: A novel AI-guided and motorized videolaryngoscope aiming to democratize endotracheal intubation
Source: Front Med (Lausanne). 2026 Feb 12;13:1744451. doi: 10.3389/fmed.2026.1744451 (PMC12937148; doi:10.3389/fmed.2026.1744451)

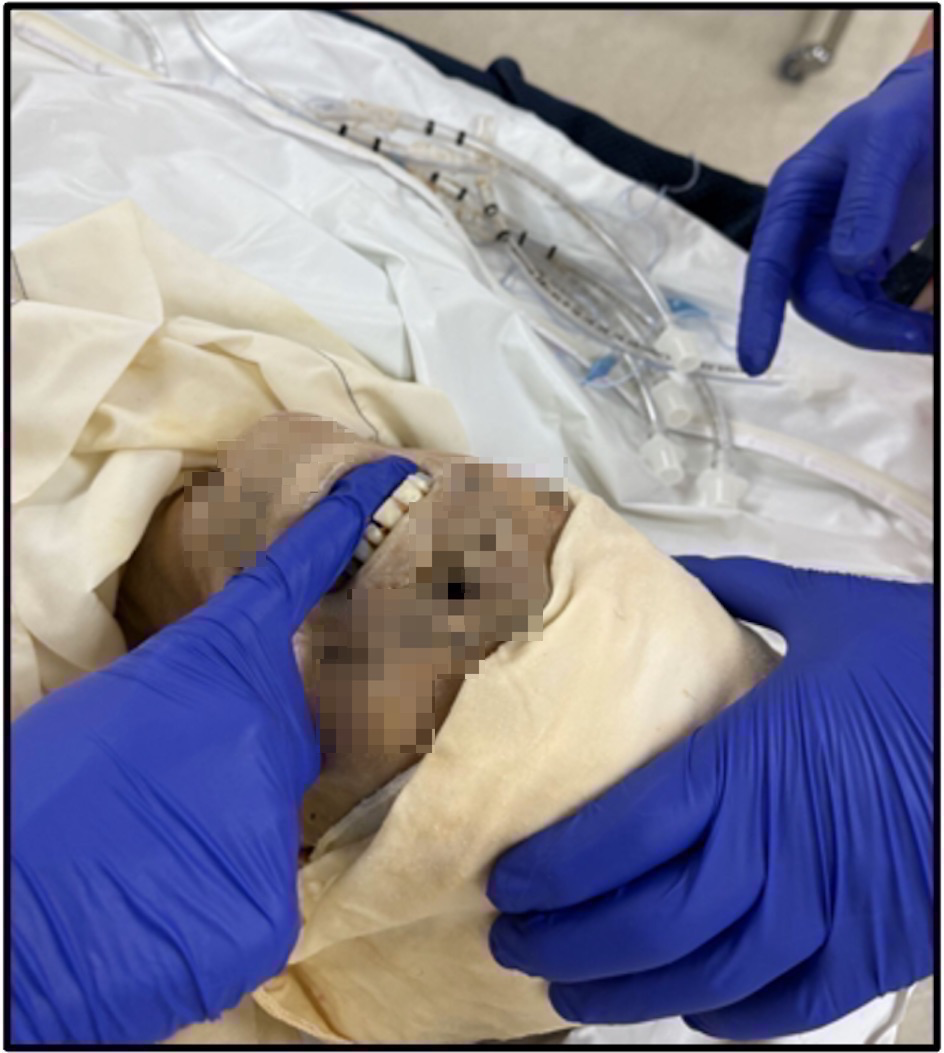

Supplement: Supplementary file 2 [file Image_1.png]
